# Resistance and virulence determinants of faecal *Salmonella* spp. isolated from slaughter animals in Benin

**DOI:** 10.1186/s13104-019-4341-x

**Published:** 2019-06-07

**Authors:** Esther Deguenon, Victorien Dougnon, Evelyne Lozes, Nana Maman, Jerrold Agbankpe, Roula M. Abdel-Massih, Fidélia Djegui, Lamine Baba-Moussa, Jacques Dougnon

**Affiliations:** 1 Research Unit in Applied Microbiology and Pharmacology of Natural Substances, Research Laboratory in Applied Biology, Polytechnic School of Abomey-Calavi, University of Abomey-Calavi, 01 PO Box 2009, Cotonou, Benin; 2 Laboratory of Biology and Molecular Typing in Microbiology, Faculty of Science and Technology, University of Abomey-Calavi, UAC, 05 PO Box 1604, Cotonou, Benin; 3 Department of Biology, Faculty of Arts and Sciences, University of Balamand, El-Koura, Lebanon; 4 Laboratory of Veterinary Diagnosis and Serosurveillance of Parakou, Ministry of Agriculture, Livestock and Fisheries, Parakou, Benin

**Keywords:** *Salmonella*, Virulence genes, Multidrug resistance

## Abstract

**Objective:**

*Salmonella* spp. are one of the leading foodborne pathogens worldwide naturally found in the intestines of many animals. People that are in direct contact with the infected animals or their cages may become ill. The aim of this study was to determine the prevalence, antibiogram and virulence genes associated with *Salmonella* serovars from fecal samples of animals intended for consumption in Southern Benin.

**Results:**

Out of a total of 406 samples, 2.46% were positive. The isolates identified were multidrug-resistant *Salmonella* spp. to penicillins, first generation cephalosporins and some aminoglycosides. All *Salmonella* isolates produced *inv*A gene of 284 bp, *fim*A of 85 bp and *stn* of 260 bp. The spvC gene (571 bp) was present in 10% of the isolates whereas the spvR gene (310 bp) was found in 20% of the isolates. The control strain possessed all the tested genes. The invA gene implies that strains are able to invade epithelial cells. The fimA and stn genes present in all isolates show that they are capable of causing gastrointestinal illness in humans. The presence of spvC and spvR genes suggests the possibility of these strains to produce toxins.

**Electronic supplementary material:**

The online version of this article (10.1186/s13104-019-4341-x) contains supplementary material, which is available to authorized users.

## Introduction

*Salmonella* is a genus of rod-shaped Gram-negative bacteria of Enterobacteriaceae family. *Salmonella* are common causes of human foodborne outbreaks in the world [1]. Every year, thousands of cases of salmonellosis-related illness and death are reported worldwide [2]. Poor hygiene standards favor the spread of *Salmonella* spp. [3]. Different serovars of *Salmonella enterica* subspecies enterica are potentially zoonotic pathogens. Different animal species, have been detected as carriers of this pathogenic agent [4]. More than 2610 *S. enterica* serovars have been recognized worldwide, being major causative agents of diseases in humans and animals [5]. Non-typhoid *Salmonella* is most often transmitted to humans through contaminated food [6].

Most cases of salmonellosis in humans are sporadic. In principle, livestock can be contaminated and therefore pose a risk to humans. The problem of the contamination of the farms is thus a concern to take into account to stop the spread of the germ. Consumption of raw or undercooked products and out-of-home catering are known risk factors, especially for *Salmonella* infections [7]. The importance of foodborne illness cannot be really estimated, but is measured in millions of annual cases [8]. Molecular characteristics of *Salmonella* in the animal population in Benin are poorly known. The present study is therefore a contribution to the knowledge of the real health risks and the exact prevalence of *Salmonella* strains of animal origin circulating in southern Benin.

## Main text

### Methods

#### Area of study

The study was conducted in southern Benin, between 6°25′N and 7°30′N and covering an area of 17,109 km^2^. The average annual temperature is 28 °C, and the humidity varies between 69 and 97% [9]. Phytogeographically, southern Benin is subdivided into four phytogeographic districts: Coastal, Pobè, Ouémé Valley and Plateau [10].

#### Fecal carriage of *Salmonella* spp. in farms and markets for slaughter meat

The collection of animal faeces, namely poultry, sheep and pigs for the search for *Salmonella* was conducted in the cities of Allada, Abomey-Calavi, Cotonou, Porto-Novo, Adjarra and Cocotomey characterized by the strong presence of breeders and large markets. Additional file 1: Figure S1 shows the cities covered by the study.

#### Period of study

The study was conducted from May to September 2018 on a sample of thirty breeding farms and nine markets. Breeding and markets selection was based on the willingness of farm owners and poultry retailers to cooperate. One visit per farm and per market was conducted. The different collections were made very early in the morning.

#### Collection of samples

Faecal samples from the available various animals in farms were collected in sterile pots using sterile swabs. The same operation was carried out in the markets. The samples were sent to the Research Unit in Applied Microbiology and Pharmacology of natural substances of the University of Abomey-Calavi for the different bacteriological analysis. The molecular identification was done at the Faculty of Arts and Sciences (University of Balamand, Lebanon) and the Laboratory of Livestock Management (Parakou, Benin).

#### Bacteriological analysis

The bacteriological analysis was conducted according to the current AFNOR standard (NF U: 47–100). Twenty-five grams of each sample were pre-enriched in 225 ml of buffered peptone water and incubated for 18 h at 37 °C. Pre-enrichment medium (0.1 ml) was then inoculated in 10 ml of selenite cystine broth for 24 h. Petri-dishes containing xylose lysine decarboxylase medium were then inoculated. Culture media were incubated at 37 °C for 24 h. Sub-cultures were done after 24 h in order to have pure colonies. After incubation at 37 °C for 24 h, the characteristic colonies of *Salmonella* spp. were considered. The urea test was performed and the colonies with this negative character were confirmed from biochemical criteria. Antibiotic susceptibility testing and molecular identification was performed for the strains identified by API20E Gallery.

#### *Salmonella* phenotypic susceptibility

An overnight bacterial pre-culture was diluted to obtain a turbidity of 0.5 McFarland (in sterile distilled water). Kirby Bauer techniques were used to perform the susceptibility testing [11]. Antibiotics of different families were chosen for the resistance pattern of the isolates: imipenem, cefotaxim, cefalotin, cefoxitin, ceftriaxone, amikacin, trimetoprim sulfamethoxazole, tobramycin, colistin, ciprofloxacin, gentamicin, nalidixic acid, chloramphenicol, amoxicillin, augmentin and fosfomicin. *Salmonella Typhimurium* ATCC 14028 was tested for quality assurance. All tests were performed in triplicates.

#### Molecular detection of virulence-associated genes

Isolates of DNA were extracted using the Qiagen blue extraction kit. The isolates were tested for different virulent genes using PCR with five sets of specific primer pairs. Several quantities of the mix were prepared (Additional file 1: Table S1).

The genes of virulence that were targeted for amplification by PCR were invA, spvR, spvC, fimA and stn (Additional file 1: Table S2).

The amplification of invA gene was carried out using the method described by Kumar et al. [12]. The amplification of spvR gene was performed using a temperature of 57 °C for 30 s [13]. fimA gene fragment was amplified at a temperature of 56 °C with extension for 30 s. spvC gene fragment was amplified at a temperature of 63 °C for 60 s. stn gene amplification was carried out at 55 °C. The amplification products were separated by 2% agarose gel electrophoresis with 5 μg/ml red gel and a 100 bp DNA ladder as a molecular weight marker. The migration was carried out at a scale of 80 V/cm for 25 min. The amplification bands were visualized and photographed under ultraviolet light (UV).

#### Statistical data processing

The data collected was coded and analyzed using Graph Pad prism 7 software.

### Results

#### Sample collection

**Fig. 1:**
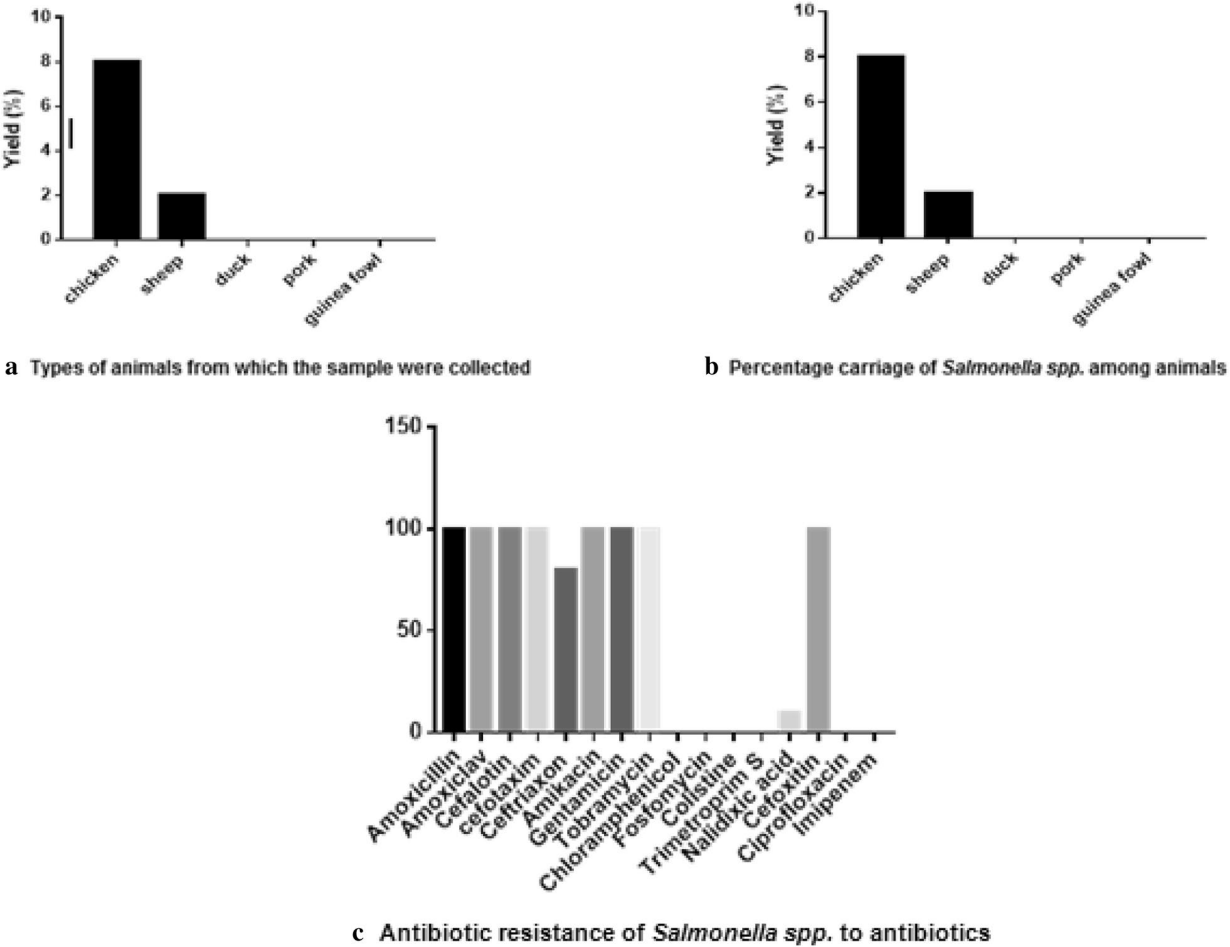
Identification of *Salmonella* spp. and resistance profile

A total of 406 samples of slaughter animal faeces were collected. Figure 1 shows the different categories of animals included in the collection.

Chicken samples were the only ones collected in the markets. A large panel of animals was obtained at the farm level. The colonies obtained after culture gave rise to the suspicion of the presence of *Salmonella* strains, especially from chickens and sheep faeces.

Chicken faeces were the most contaminated with *Salmonella* spp. (Fig. 1). Additional file 1: Figure S2 shows the appearance of a faecal sample inoculated on XLD agar.

The colonies characterize *Salmonella* colonies on this selective medium. The isolates were then purified and identified by API20E Gallery. The prevalence of *Salmonella* isolated from faeces of slaughter animals was 2.46% during the period from May to September 2018.

#### Susceptibility test of *Salmonella* strains

Figure 1 shows the resistance profile of *Salmonella* spp. identified. Strains were resistant to all penicillins, aminoglycosides and to first and second generation cephalosporins. 10% of the strains were resistant to fluoroquinolones. Additional file 1: Figure S3 shows inhibition zone of antibiotics on isolated *Salmonella* species. *Salmonella* has natural resistance to generation one and two of cephalosporins. Aminoglycosides were tested only as reference and not meant for clinical treatment since susceptibility does not reflect in vivo only in vitro activity [14]. Additional file 1: Table S3 shows the antibiotic susceptibility patterns (%) of isolates strains of *Salmonella* spp.

#### Molecular identification

All *Salmonella* isolates were positive for the presence of invA genes (284 bp), fimA (85 bp) and stn (260 bp). The spvC gene was present in 10% and spvR gene (310 bp) in 20% of the isolates. The reference strain was positive for all genes and served as control. Table 1 shows the genes isolated from each isolate of *Salmonella* spp.

**Table 1:** Virulence genes identified

Strains	Virulence genes				
	*inv*A	*spv*R	*Spv*C	*Fim*A	*Stn*
P_9_	+	+		+	+
P_14_	+			+	+
P_15_	+			+	+
P_16_	+			+	+
P_17_	+			+	+
P_19_	+	+	+	+	+
P_29_	+			+	+
P_70_	+			+	+
P_362_	+			+	+
P_368_	+			+	+
*S. Typhimurium* ATCC 14028	+	+	+	+	+

**Fig. 2:**
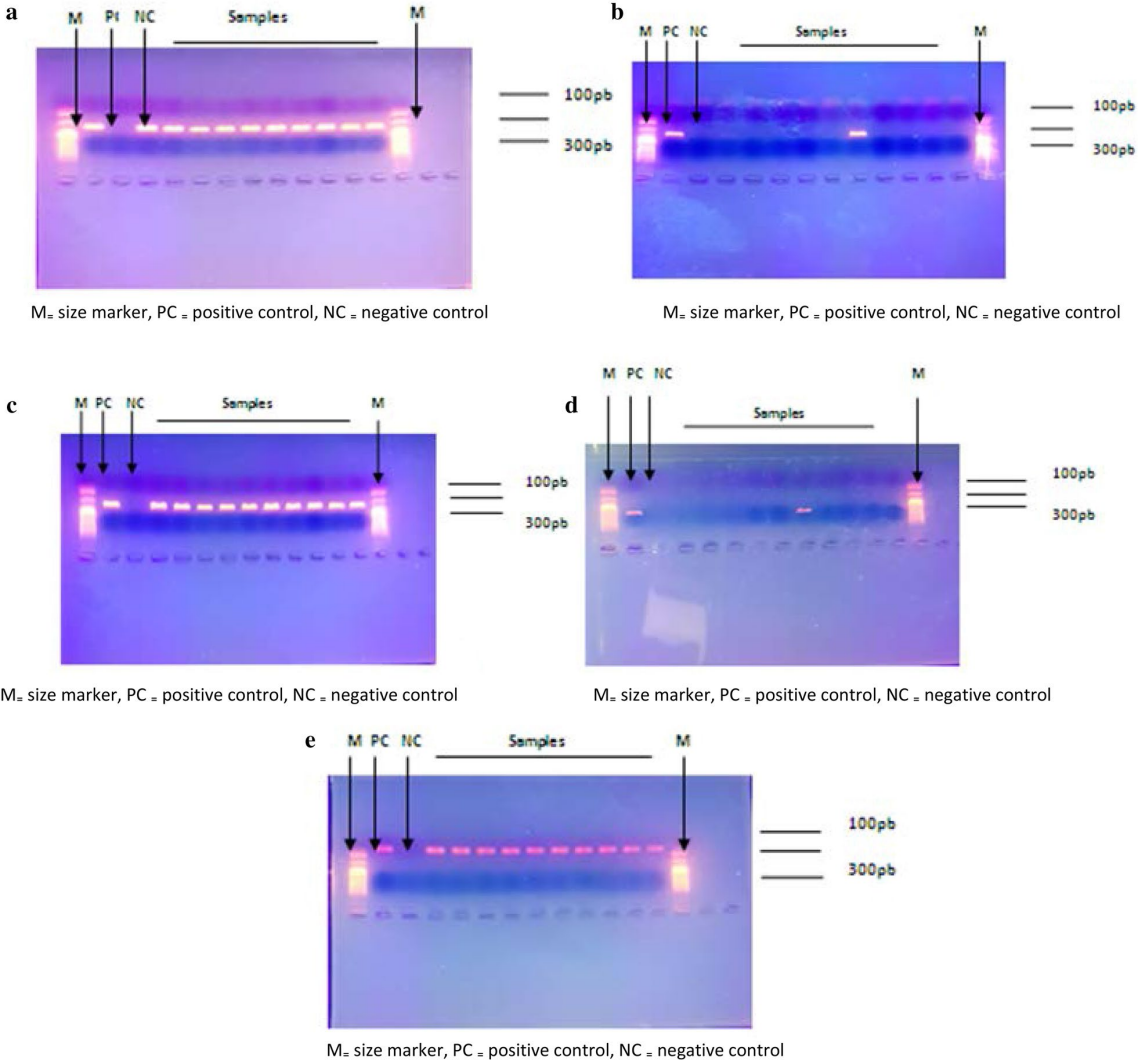
Agarose gels after PCR. gel. **a** invA genes (284 bp), **b** spvR gene (310 bp), **c** spvC (571 bp), **d** fimA (85 bp), **e** stn

*Salmonella* strain P_19_ found in faeces of local hens sold on the market was the only with all the virulences genes. Figure 2 shows the agarose gels after PCR.

### Discussion

The study carried out revealed the prevalence of virulence genes of circulating strains in Southern Benin. It should be noted that *Salmonella* are mostly used as markers of biological risk [15]. From the results obtained, a prevalence of 2.46% *Salmonella* spp. was isolated from market poultry faeces and from farm sheep. This prevalence is low compared to 20% of René et al. [16] in Abidjan and 3.6% found by Barilli et al. [17] in Northern Italy. However, in the studies mentioned, only pigs and cattle were considered respectively. The low prevalence found in the present study can be attributed to climate conditions. Indeed, sample collection was made in dry season characterized by humidity absence. The antimicrobial resistance profile showed resistance of all strains to penicillins, to first and second generation cephalosporins, to aminoglycosides and partially to fluoroquinolones. These results are contradictory to those of Dong et al. [18] which revealed a sensitivity of *Salmonella* strains to penicillins. Other studies have also reported the problem of multiresistance of *Salmonella* spp. [19]. The importance of *Salmonella* spp. as potentially dangerous bacteria can be influenced by both multidrug resistance and presence of virulence genes. *Salmonella enterica* has several pathogenicity islands in its genome, which are genetic elements that harbor genes associated with virulence [20]. The results obtained after PCR confirmed the presence of invA, spvR, spvC, fimA and stn genes. The presence of invA in all isolates proves that they have a potentially invasive power. Chaudhary et al. [21] reported similar results. Oliveira et al. [22] reported that the search for invA, specific for *Salmonella* spp. significantly reduces the number of false negatives that occur in laboratory diagnostics. The amplification of invA is currently recognized as international standard for the detection of *Salmonella* [23]. The spvR gene present in 20% of the strains, gives them capacity to cause systemic infections. These results are consistent with those of Chaudhary et al. [21] and Araque [24].

The spvC gene was present in 10% of the isolates. This gene is able to inhibit the activation of macrophages and initiate their apoptosis [25]. The results obtained are similar to those of Bolton et al. [26]. On the other hand, Chaudhary et al. [21] reported the total absence of this gene in all isolates. This gene is therefore not systematically found in the *Salmonella* genome but is of paramount importance when present. Kryzanowski et al. [27] found a low rate of *Salmonella* strains possessing spvC gene, suggesting his particularity in the virulence of *Salmonella*. The rarity of spv genes in the *Salmonella* genome has also been demonstrated in other studies, which have revealed that they are responsible for the systemic infection and multidrug resistance in humans and animals [28]. They are also involved in intracellular bacterial proliferation [29]. The search for the presence of spv genes can increase the possibility of *Salmonella* strains to be of significant clinical interest [30]. As for fimA gene, its presence indicates the presence of fimbriae, important factor for *Salmonella* to adhere to epithelial cells. This result is similar to those of Boriello et al. [31]. Similar to the work of Barilli et al. [14], the stn gene was present in all isolates. Nakano et al. [32] revealed that stn is suspected to contribute to enterotoxigenic potency.

### Conclusion

The presence of multidrug resistant *Salmonella* spp. in the faeces of animals is of major concern and the presence of virulence genes confirms the possible pathogenicity of these strains. The present study is therefore of paramount importance in the surveillance of salmonellosis.

## Limitations

Sequencing of *Salmonella* genome is not possible in Benin.

## Additional file

**Additional file 1: Figure S1.** Map of Southern Benin showing cities covered by the study. **Figure S2.** Appearance of *Salmonella* strains on XLD medium. **Figure S3.** Inhibition zones of different antibiotics on isolated strains of *Salmonella* spp. **Table S1.** PCR Reaction Medium. **Table S2.** Resistance genes. **Table S3.** Resistance profile of *Salmonella* spp. strains against antibiotics.

## Data Availability

All data generated or analysed during this study is included in this published article and Additional file.
