# Supplementary material for: Resistance and virulence determinants of faecal Salmonella spp. isolated from slaughter animals in Benin
Source: BMC Res Notes. 2019 Jun 7;12:317. doi: 10.1186/s13104-019-4341-x (PMC6556020; doi:10.1186/s13104-019-4341-x)
Supplement: Supplementary file 1 — Additional file 1: Figure S1. Map of Southern Benin showing cities covered by the study. Figure S2. Appearance of Salmonella strains on XLD medium. Figure S3. Inhibition zones of different antibiotics on isolated strains of Salmonella spp. Table S1. PCR Reaction Medium. Table S2. Resistance genes. Table S3. Resistance profile of Salmonella spp. strains against antibiotics. [file 13104_2019_4341_MOESM1_ESM.docx]

**Additional file 1**


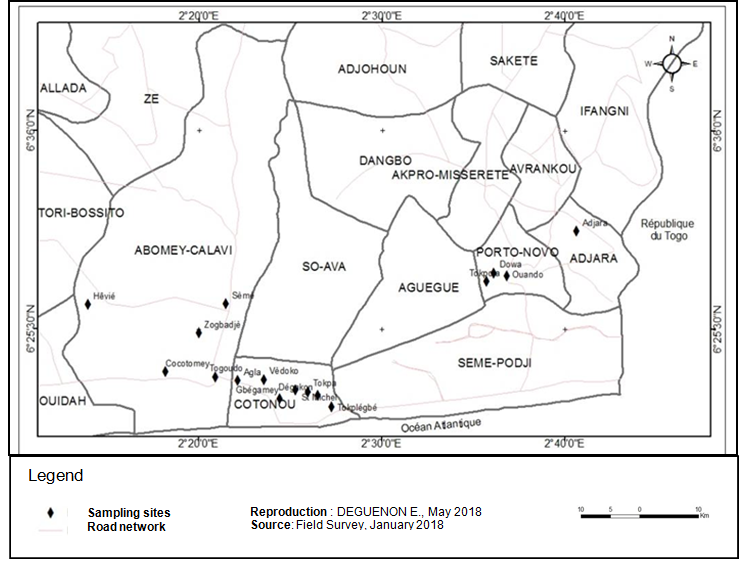


**Figure S1: Map of Southern Benin showing cities covered by the study**


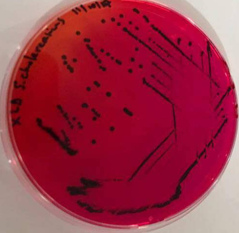


**Figure S2:** Appearance of *Salmonella* strains on XLD medium


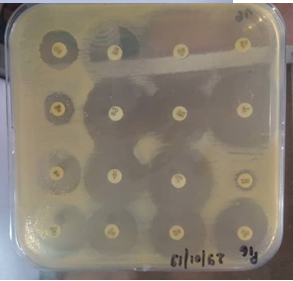


**Figure S3:** Inhibition zones of different antibiotics on isolated strains of *Salmonella spp*.

**Table S1:** PCR Reaction Medium

| **Components** | **Volume (µl)** |
| --- | --- |
| Water | 7.5 |
| 2XPCR Master Mix | 12.5 |
| Primer-F (10µmol/μl) | 1 |
| Primer-R (b) (10µmol/μl) | 1 |
| DNA | 3 |
| Total | 25 |

**Table S2:** Resistance genes

| **Primers** | **Sequences** | **References** |
| --- | --- | --- |
| INVAF | GTGAAATTATCGCCACGTTCGGGCAA | [12] |
| INVAR | TCATCGCACCGTCAAAGGAACC |  |
| SPVRF | CAGGTTCCTTCAGTATCGCA | [13] |
| SPVRR | TTTGGCCGGAAATGGTCAGT |  |
| SPVCF | ACTCCTTGCACAACCAAATGCGGA | [14] |
| SPVCR | TGTCTTCTGCATTTCGCCACCATCA |  |
| FIMAF | CCTTTCTCCATCGTCCTGAA | [15] |
| FIMAR | TGGTGTTATCTGCCTGACCA |  |
| STNF | CTTTGGTCGTAAAATAAGGCG | [16] |
| STNR | TGCCCAAAGCAGAGAGATTC |  |

**Table S3: Resistance profile of *Salmonella spp* strains against antibiotics**

|  | **Antibiotic susceptibility patterns (%)** | | | | | | | | | | | | | | | |
| --- | --- | --- | --- | --- | --- | --- | --- | --- | --- | --- | --- | --- | --- | --- | --- | --- |
|  | **AMX** | **AMC** | **CF** | **FOX** | **CTX** | **CRO** | **AN** | **CN** | **NM** | **C** | **FOS** | **CL** | **SXT** | **NA** | **CIP** | **IPM** |
| **Susceptible** | 0 (0) | 0 (0) | 0 (0) | 0 (0) | 0 (0) | 9 (90) | 0 (0) | 0 (0) | 0 (0) | 10 (100) | 10 (100) | 10 (100) | 10 (100) | 9 (90) | 10 (100) | 10 (100) |
| **Resistance** | 10 (100) | 10 (100) | 10 (100) | 10 (100) | 10 (100) | 1  (10) | 10 (100) | 10 (100) | 10 (100) | 0  (0) | 0  (0) | 0  (0) | 0  (0) | 1  (10) | 0  (0) | 0  (0) |
| **Total** | 10 | 10 | 10 | 10 | 10 | 10 | 10 | 10 | 10 | 10 | 10 | 10 | 10 | 10 | 10 | 10 |

AMX = Amoxicillin, AMC = Amoxicillin clavulanic acid CF = Cefalotin, FOX = Cefoxitin, CTX =Cefotaxime, CRO = Ceftriaxone, AN = Amikacin, CN = Gentamycin, NM = Tobramycin, C = Chloramphinicol, FOS = Cefoxitin, CL = Colistin, , SXT =Trimetroprim sulfametozaxol, NA = Nalidixic acid Ceftriaxone, CIP = Ciprofloxacin, , IPM = Imipenem

**References**:

15. Naravaneni R, Jamil K. Rapid detection of food-borne pathogens by using molecular techniques. J Med Microbiol. 2005;54:51–4.

16. Makino S, Kurazono H, Chongsanguam M, Hayashi H. Establishing of the PCR system specific to *Salmonella* spp. and its application for the inspection of food and fecal samples. J Vet Med Sci. 1999;61(11):1245–7.

https://doi.org/10.1292/jvms.61.1245
